# *QuickStats:* Percentage of Adults Aged 18–64 Years with Current Asthma,* by State — National Health Interview Survey,^†^ 2014–2016

**DOI:** 10.15585/mmwr.mm6720a7

**Published:** 2018-05-25

**Figure:**
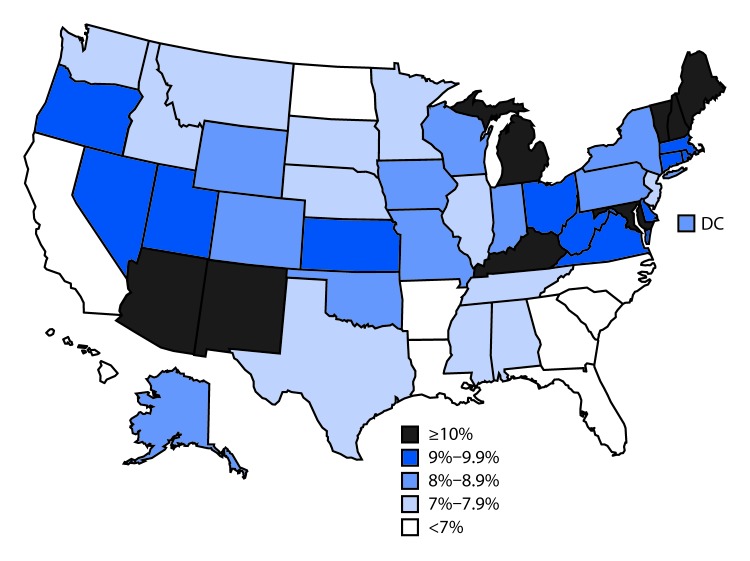
During 2014–2016, 8% of U.S. adults aged 18–64 years had current asthma. Current asthma prevalence was highest in New Hampshire (12.7%), Vermont (12.3%), Arizona (11.0%), Kentucky (10.8%), and Maine (10.8%). The prevalence was lowest in Hawaii (4.9%), North Dakota (5.7%), Arkansas (5.9%), South Carolina (6.2%), and North Carolina (6.2%).

**Source: **National Health Interview Survey, 2014–2016. https://www.cdc.gov/nchs/nhis.htm. Tabular results available at https://www.cdc.gov/nchs/data/health_policy/asthma_table_SEs.pdf.

